# A Comparative Study on Ascorbic Acid Concentration, Total Phenol, and Flavonoid Content in Citrus Species Grown in a Different Region of Western Nepal

**DOI:** 10.1155/2022/3012623

**Published:** 2022-12-19

**Authors:** Roshani Gurung, Sabina Baral, Santosh Parajuli, Dhana Dhami, Sushila Ghimire

**Affiliations:** ^1^Department of Pharmacy, Shree Medical and Technical College, Affiliated to Purbanchal University, Bharatpur-12, Chitwan 44200, Nepal; ^2^Pharmacy Programme, Gandaki University, Pokhara, Kaski, 33700, Nepal

## Abstract

Different genetic and environmental factors like altitude, temperature, light radiation, etc. affect the production of phytoconstituents like ascorbic acid, phenol, flavonoid, tannin, etc. So, it is necessary to evaluate phytoconstituents quantitatively and qualitatively because of their different medicinal values. This study is aimed at evaluating and comparing ascorbic acid concentration, total phenol, and flavonoid content in citrus species grown in different places in Western Nepal. For this, fruit collected from Lamjung and Nawalparasi was peeled, and collected juice was extracted with ethanol. Ascorbic acid was estimated using titration with dichlorophenolindophenol dye, and total phenol was estimated using Folin reagent; whereas, flavonoid content was determined by aluminum chloride colorimetric assay. The result showed the maximum ascorbic acid concentration in the fruit juice of *Citrus maxima* collected from Lamjung, i.e., 2.98 ± 0.161 mg/100 ml, and the least concentration was recorded on extract of *Citrus limon* collected from Nawalparasi, i.e., 1.005 ± 0.205 mg/100 ml. The total phenol content was comparatively higher in *Citrus maxima* and *Citrus aurantifolia* collected from Lamjung, i.e., 12.48 ± 0.40 mg GAE/gm, respectively. Flavonoid content was comparatively higher in *Citrus maxima* collected from Nawalparasi, i.e., 484.466 ± 3.055 mg QE/gm. This study showed a variation in the concentration of chemical constituents between different places in Western Nepal. This might be due to differences in climatic conditions, environmental factors, altitude, temperature, etc. This study helps to reflect the best-suited altitude for commercial cultivation of the citrus species as these phytochemicals have different medicinal values. Also, this study can help the food industry to use an alternative source of synthetic antioxidant such as ascorbic acid, phenol, and flavonoid. And local people will economically benefit by the commercial cultivation of citrus fruits.

## 1. Introduction

Plants are a good source of phytochemicals such as phenol, flavonoid, alkaloids, sterols, terpenoids, phenolic acids, stilbenes, lignans, tannins, and saponins, and some plant also contains vitamin C (ascorbic acid), vitamin E, and carotene [1]. Due to the presence of these phytochemicals, plants show many pharmacological activities such as antioxidant activity, wound healing properties, anti-inflammatory activity, pain healing, antidiarrheal activity, antimicrobial, and anticancer activity [2, 3]. Citrus fruits are an important source of different bioactive compounds such as ascorbic acid, flavonoid, and phenol, which have antioxidant activity [4–7]. Many studies have suggested that increased dietary intake of natural phenolics correlates with reduced coronary heart disease and cancer mortality with longer life expectancy and also found effective in many health-related properties, such as antioxidant, anticancer, antiviral, and anti-inflammatory activities [8]. Vitamin C (ascorbic acid) also have used for many purposes like nutrition, antioxidant activity, cosmetic purpose, fat burning, menstrual disorders, diarrhea, dysentery, insomnia, sedation, asthma, scurvy, gout, and arthritis. It helps to lower the cholesterol level in the blood and cures ulcers [9, 10]. Previously, it was reported that phytochemical profiles and antiradical scavenging activity may significantly vary among citrus species, among cultivars within the same species, and even within the same cultivar grown in diverse climatic conditions [10]. Because many factors affect the production of phytoconstituents as genetic and environmental factors, i.e., climatic conditions, soil nutrition, altitude, atmospheric pressure, temperature, light, maturity stage, amount of nitrogen fertilizer used, the position of fruit on the tree where it is present on the tree, duration of storage, etc. [11], it became important to determine the concentration of phytoconstituents quantitatively and qualitatively because citrus fruits have an important role in our health and nutrition.

Many methods can be used for the quantitative determination of ascorbic acid, total phenol, and flavonoid content. Previously, the determination of ascorbic acid concentration was done by spectrophotometry, titrimetric method, liquid chromatography, and capillary electrophoresis [12, 13].

In this study, three citrus fruits (*Citrus limon*, *Citrus maxima*, and *Citrus aurantifolia*) growing in different regions of Western Nepal, i.e., Lamjung having an altitude of 760 m and Nawalparasi having an altitude of 300-400 m, have been selected for comparative analysis of different phytoconstituents (ascorbic acid, total phenol, and flavonoid content).

Few studies have been done in Nepal on citrus fruit focusing on ascorbic acid concentration to date [14], but no study has been done on a comparison of chemical variation in citrus fruit grown in different regions of Western Nepal since the phytochemical variation is dependent on different environmental factors like temperature, light, soil conditions, rainfall, altitude, genetic factor, etc. It will help to recognize the suitable environmental condition and storage conditions for the commercial cultivation of citrus fruits because the production of phytoconstituents is affected by environmental factors. This study is important for exploring the quantitative variation of ascorbic acid, total phenol, and flavonoid content from a different region of Western Nepal, about which only limited study is available. So, ultimately, it can be expected that local people will be benefited economically from the commercialization of cultivation which can also derive an idea about the best quality of citrus fruit that benefits our health consisting of higher concentration of ascorbic acid, phenol, and flavonoid which is responsible for many pharmacological activities. Also, this study can help the food industry to use ascorbic acid, phenol, and flavonoid as an alternative to synthetic antioxidants since it is environmentally friendly and safe for consumption.

## 2. Materials and Methods

### 2.1. Chemicals and Equipment/Instruments

All chemicals used were analytical grade reagents, and all required chemicals were obtained from Shree Medical and Technical College (SMTC), Bharatpur, Chitwan which was purchased from authorized suppliers through the laboratory of Shree Medical and Technical College, i.e., aluminum chloride (Qualikems Fine Chem, Gujarat, India), ascorbic acid, dichlorophenolindophenols, folin-ciocalteu reagent, ethanol, gallic acids, oxalic acid, quercetin, sodium bicarbonate, sodium hydroxide, and sodium nitrate (Medica Enterprise Pvt. Ltd, Punjab, India). List of the instrument/equipment used for the study is spectrophotometer (LT-2900, Labtronics, Haryana, India), hot air oven (Shiv Enterprises, Ghaziabad, India), water bath (Shiv Enterprises, Ghaziabad, India), and weighing balance (ALJ, Kern and Sohn, Germany).

### 2.2. Study Site

Citrus fruits (*Citrus limon*, *Citrus maxima*, and *Citrus aurantifolia)* were selected for the study because they contain higher concentration of ascorbic acid, phenol, and flavonoid than other citrus fruits. For collection of samples, different regions of Western Nepal, i.e., Lamjung having an altitude 760 m and Nawalparasi having an altitude 300-400 m, were selected because these samples are mostly found in these two areas.

### 2.3. Collection and Authentication of Plant Material

Citrus fruits (*Citrus limon*, *Citrus maxima*, and *Citrus aurantifolia)* were collected from different places of Western Nepal, i.e., from Lamjung having an altitude of 760 m and another from Nawalparasi having an altitude of 300-400 m and then identified by botanist Mr. Bishnu Prasad Bhattarai, Birendra Multiple Campus, Chitwan, Nepal. Then, samples were deposited in Pharmacognosy Lab of Shree Medical and Technical College, Chitwan for further references.

### 2.4. Preparation of Extract

Fresh fruits were peeled off and squeezed to obtain juices. From that, 50 gm of juice was kept in a round bottle flask having 500 ml of 99.9% ethanol and was boiled on a water bath for 2 hr at temperature of 70°C. Then, filtration was done followed by solvent evaporation in heating mantle until brown-red viscous extract was obtained and was stored at the refrigerator for further use in experiments, and the yield value of extract was calculated by using the following formula as [15]

(1) $$\begin{array}{c} \%\text{Extractive value}\left(\frac{W}{W}\right)=\frac{\left(\text{Final weight of extract}\right)}{\left(\text{Weight of juice}\right)}*100\%. \end{array}$$

### 2.5. Estimation of Ascorbic Acid

#### 2.5.1. DCIP Titration Method

Ascorbic acid was determined according to the method of [16] with some modification. Then, each extract containing 0.1 gm of citrus fruits was weighed, and 20 ml of 4% oxalic acid was added to each of the extracts. The mixture was well shaken and left for 15 min. From this, a 10 ml sample's aliquot and 10 ml of 4% oxalic acid were taken in a conical flask. This was then titrated against the DCIP dye of the burette, and the appearance of pink color was taken as an endpoint (*V*2). This process was repeated thrice for each extract. The amount of ascorbic acid was calculated using the formula [16, 17].

(2) $$\begin{array}{c} \text{Ascorbic acid }\left(\frac{mg}{g}\right)=\frac{0.5*V2*20}{V1*10*0.1}, \end{array}$$

where *V*2 is the volume of sample after titration, *V*1 is the volume of standard after titration, 20 is the volume of content in conical flask, 10 is the volume of test solution, 0.1 is the weight of sample in gm, and 0.5 is the minimum value of concentration.

### 2.6. Estimation of Total Phenol Content

Gallic acid was taken as a standard drug for the estimation of total phenol content in citrus fruits. Folin-Ciocalteu method was used for the determination of total phenol content according to the method of [18] with some modifications. The total phenol content was expressed as mg of GAE (gallic acid equivalent) per g dry extract weight using the calibration curve of gallic acid from the standard solution of gallic acid, 50 mg/l, 100 mg/l, 200 mg/l, 300 mg/l, 400 mg/l, and 500 mg/l. In brief, 1 ml of sample was mixed with 5 ml of distilled water and 1 ml of folin reagent. After standing for 5 min, 1 ml of 10% sodium carbonate was added and stirred. The mixture was incubated for 1 hr at room temperature, and the absorbance was measured at 725 nm against a blank.

### 2.7. Estimation of Total Flavonoid Content

Quercetin was used as standard, and flavonoid content was determined and expressed as quercetin equivalent from the calibration curve of the dilutions of 50 mg/l, 100 mg/l, 200 mg/l, 300 mg/l, 400 mg/l, and 500 mg/l. The total flavonoid content of various fruits extract was determined by aluminum chloride complex-forming assay as described previously by [18], with some modifications. In brief, 1 ml of the sample solution was mixed with 4 ml of distilled water. Then, 300 *μ*l of sodium nitrite was added. After 5 min, 300 *μ*l aluminum chlorides were added and allowed to stand for 6 min. Then, 2 ml of sodium hydroxide was added. The mixture was stirred, and the absorbance was measured at 510 nm using UV spectrophotometer and compared with standard.

### 2.8. Statistical Analysis

The data obtained during the experiment were expressed as mean ± standard deviation.

## 3. Results and Discussion

### 3.1. Extractive Value

The yield value of ethanolic extract of all citrus fruit has been shown in Table 1.

### 3.2. Ascorbic Acid Content

Maximum ascorbic acid content was observed in fruit juice of *Citrus maxima* collected from Lamjung, i.e., 2.98 ± 0.161 mg/100 ml and the least on an extract of *Citrus limon* collected from Nawalparasi, i.e., 1.005 ± 0.205 mg/100 ml. The result of ascorbic acid content in citrus species has been shown in Table 2.

### 3.3. Total Phenol Content

**Figure 1:**
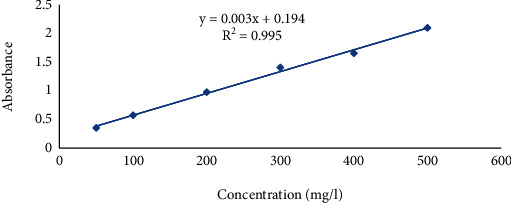
Calibration curve of gallic acid for the estimation of total phenol content.

The calibration curve of standard gallic acid has been shown in Figure 1. The results showed that the sample collected from Lamjung, i.e., *Citrus aurantifolia* and *Citrus maxima*, contains higher phenol content, i.e., 12.48 ± 0.400 mg GAE/gm and 12.48 ± 0.400 mg GAE/gm, respectively. *Citrus limon* collected from Nawalparasi showed the lowest phenol content, i.e., 2.66 ± 0.400 mg GAE/gm. The result of the total phenol content has been shown in Table 3.

### 3.4. Total Flavonoid Content

**Figure 2:**
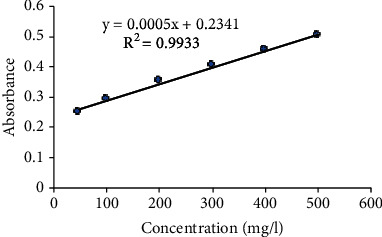
Calibration curve of quercetin standard for estimation of flavonoid content.

The calibration curve of standard sample quercetin has been shown in Figure 2 showing *R*^2^ = 0.9933. The samples collected from Nawalparasi, i.e., *Citrus aurantifolia* and *Citrus maxima*, were found to contain the higher flavonoid content than the sample collected from Lamjung, i.e., 385.8 ± 2.000 mg QE/gm and 484.46 ± 3.055 mg QE/gm, respectively. But, the sample *Citrus limon* collected from Lamjung showed high flavonoid content, i.e., 381.13 ± 3.055 mg QE/gm than the sample collected from Nawalparasi. The result has been shown in Table 4 and Figure 2.

## 4. Discussion

From this study, we observed quantitative variation of phytoconstituents (ascorbic acid, total phenol, and flavonoid content) in citrus species grown in different regions of Western Nepal. Phytoconstituents, like ascorbic acid flavonoids, phenols, alkaloids, etc., are being used in allopathic and also found to be used traditionally as ethnomedicines for antiviral properties like in COVID-19 treatment [19–21]. The extraction of the juice of citrus fruit was done using ethanol as a solvent, and the extractive value was found to be higher in citrus species collected from Nawalparasi. Higher extractive value might be due to the presence of more ethanol-soluble chemical constituents, i.e., tannin, terpenoids, and hesperidin which might be less in citrus fruit collected from Lamjung [22].

Ascorbic acid is also known as vitamin C, an essential nutrient that plays a vital role in protecting the body from infection and diseases, and is also important for the proper function of the immune system, and it is present in higher concentrations in citrus fruits [23]. In this study, ascorbic acid was determined both in juice and extract. The ascorbic acid concentration in fruit juice was considerably low as compared to those obtained by the previous study; such variation might be due to differences in nature, the origin of species, and the solvent used for the extraction method [17]. Result clearly shows that the ascorbic acid content in the extract was less as compared to fresh juice. The ascorbic acid content in the juice was higher in citrus species collected from Lamjung at a higher altitude (760 m) than the sample collected from Nawalparasi at a lower altitude (300-400 m), and it might be due to fruit position on the tree, climatic/environmental conditions, ripening stage, and species and variety of the citrus fruits as well as temperature; the ascorbic acid content of citrus fruits is never stable but varies. As well as different techniques of measuring and squeezing technique, duration and storage may also affect the concentration of ascorbic acid [14, 24]. These findings were supported by the previous study done previously [25–28]. A decrease in ascorbic acid could be due to enzymatic loss of L-ascorbic acid where it is converted to 2-3-deoxy-L-gluconic acid [29].

Phenol is commonly present in fruits, vegetables, leaves, nuts, seeds, barks, roots, etc. They act as reducing agents, hydrogen donors, singlet oxygen quenchers, and metal chelators [11, 30]. The total phenol content of juice samples was quantified by folin-ciocalteu assay which depends on the reduction of folin-ciocalteu reagent by phenolic compounds under alkaline conditions. Absorbance is directly proportional to the concentration of phenolic compounds, which is represented by the intensity of the blue color produced in each solution [31].

The total phenol content was higher in *Citrus limon*, *Citrus aurantifolia*, and *Citrus maxima* collected from Lamjung. This study was supported by previous studies [32–34]. The increase in altitude may cause a decrease in temperature and ascribe as a response of plants to radiation which causes amplified biosynthesis of UV-absorbing and enhanced UV-B antioxidant phenolics in plants. And in a recent study, it was found that there was a significant increase in the biosynthesis of phenolics in plants growing in a low-temperature region [35, 36].

Flavonoids are found in fruits, vegetables, grains, bark, roots, stem, flowers, tea, and wine, and it has been found that citrus flavonoids are present in almost all the parts of citrus fruits in different species Previous studies have reported that flavonoid possesses different pharmacological activities such as antioxidant, anti-inflammatory, analgesia, and the antinociceptive effect [37]. Flavonoid content in citrus fruits was measured by aluminum chloride colorimetric assay using quercetin as standard. The flavonoid in the presence of aluminum chloride has intense yellow fluorescence which is observed in the UV spectrometer at 510 nm [12]. Flavonoid content on *Citrus limon* collected from Nawalparasi was less than that collected from Lamjung; whereas, *Citrus maxima* and *Citrus aurantifolia* collected from Nawalparasi showed a higher concentration of flavonoid as compared to that of Lamjung which might be due to the presence of thermal resistant flavonoid. The total flavonoid content of citrus fruits in this study was comparatively higher than those obtained by the previous study on fresh juices of *Citrus hystrix* and *Citrus maxima* [38]. It was found that the concentration of flavonoids on the peel was usually higher than in the tissue of citrus species [39]. Hence, the variation of chemical constituents depends on many environmental factors, genetic factors, hormonal factors, age of the plant, most citrus species accumulate substantial quantities of flavonoids during organ development, maturity of the fruit, the postharvesting treatments, and the extracting processes [40]. Similar findings were observed in previous studies [33, 41]. Thus, it was concluded that the variety and stages of ripening and environmental factor had a significant effect on total flavonoids, total polyphenols, and antioxidant activity [42].

## 5. Conclusion

The study showed that the ascorbic acid concentration of citrus fruits in the juice was comparatively higher than in juice extract. Climatic conditions, altitude, temperature, environmental factors, variety, and ripening stage affect the chemical constituents present in citrus fruits. It also concluded more content of ascorbic acid concentration, total phenolic content, and flavonoid and is found in higher altitudes, with low temperatures, i.e., Lamjung (760 m), than a plant grown in higher temperatures. This study also helps to know the best-suited environmental condition for commercial cultivation of the species as these phytochemicals have medicinal value, and the food industry can use ascorbic acid, phenol, and flavonoid as an alternative to synthetic antioxidants since it is environmentally friendly and safe for consumption. Also, this study helps to grow the economic standard of local people through commercial cultivation and production of citrus fruits. In the future, further study should be done to know the exact reason for variation in phytoconstituents in citrus fruit also needed for evaluation and comparison of biological activity in these citrus fruits.

## Figures and Tables

**Table 1:** Extractive value of citrus fruits.

Citrus fruits	Lamjung	Nawalparasi
*Citrus lemon* (Kagati)	7.96%	10.84%
*Citrus aurantifolia* (Nibuwa)	5.96%	6.04%
*Citrus maxima* (Bhogate)	6.56%	12.46%

Note: Extractive value was calculated by using Equation (1).

**Table 2:** Ascorbic acid content in fresh juice and extract.

S.N.	Sample	Collection site	Ascorbic acid (juice) (mg/100 ml)	Ascorbic acid (extract) (mg/100 ml)
1.	*Citrus Limon*	Lamjung	2.69 ± 0.167	1.44 ± 0.000
		Nawalparasi	2.11 ± 0.160	1.005 ± 0.205

2.	*Citrus aurantifolia*	Lamjung	1.92 ± 0.169	1.876 ± 0.207
		Nawalparasi	1.73 ± 0.290	1.0085 ± 0.200

3.	*Citrus maxima*	Lamjung	2.98 ± 0.161	2.165 ± 0.205
		Nawalparasi	2.31 ± 0.000	2.885 ± 0.813

Note: Ascorbic acid content was determined by using Equation (2). Each experiment was done triplicate.

**Table 3:** Total phenol content in citrus fruit extract.

Place	*Citrus limon* (mg GAE/gm)	*Citrus aurantifolia* (mg GAE/gm)	*Citrus maxima* (mg GAE/gm)
Lamjung	9.94 ± 0.520	12.48 ± 0.400	12.48 ± 0.400
Nawalparasi	2.66 ± 0.400	9.33 ± 0.400	2.11 ± 0.520

Note: Data expressed as mean ± standard deviation. Each experiment was done triplicate.

**Table 4:** Flavonoid content in fruit extract.

Sample collection site	*Citrus limon* (mg QE/gm)	*Citrus aurantifolia* (mg QE/gm)	*Citrus maxima* (mg QE/gm)
Lamjung	381.13 ± 3.050	328.46 ± 3.055	474.46 ± 3.055
Nawalparasi	313.8 ± 2.000	385.8 ± 2.000	484.46 ± 3.055

Note: Data expressed as mean ± standard deviation. Each experiment was done triplicate.

## Data Availability

The data used to support the findings of this study are included within the article.

## Abbreviations

wt: Weight
mg: Milligram
g: Gram
ml: Milliliter
DCIP: Dichlorophenolindophenol
GAE: Gallic acid Equivalent
QE: Quercetin Equivalent
UV: Ultraviolet
PU: Purbanchal University
SMTC: Shree Medical and Technical College.
